# Sibling Support Program: A Novel Peer Support Intervention for Parents, Caregivers and Siblings of Youth Experiencing Mental Illness

**DOI:** 10.3390/healthcare10050908

**Published:** 2022-05-13

**Authors:** Joshua Feriante, Ariella Shayani, Emily Lauer, Adele Pressman, Emily Rubin

**Affiliations:** 1 Department of Psychiatry, UMass Chan Medical School, 55 N Lake Avenue, Worcester, MA 01655, USA; joshua.feriante@umassmemorial.org (J.F.); ariella.shayani@umassmemorial.org (A.S.); emily.lauer@umassmed.edu (E.L.); 2 Cambridge Health Alliance, 1493 Cambridge Street, Cambridge, MA 02139, USA; adpressman@challiance.org

**Keywords:** sibling support, peer support, family support, family-centered mental health care

## Abstract

Caregivers and siblings of youth with mental illness often experience role-related psychological challenges, and it is important to focus on the needs of these family members. Existing literature demonstrates that caregivers and affected children benefit from participation in peer support and family-centered programs. This paper describes the Sibling Support Program: A Family-Centered Mental Health Initiative (SSP), a novel intervention for families of youth with mental illness. The SSP distinguishes itself from existing family-centered programs in that it utilizes a unique combination of peer support, parent mentor guidance, and clinician-led group therapy. The paper details the structure of the treatment model and presents preliminary data from participant surveys. Results show preliminary indications that the program provides both emotional and practical benefits. Along with high satisfaction ratings, family members report decreased feelings of isolation, gains in knowledge, and more positive thinking after program participation. Caregivers report that the SSP helped improve their understanding about the impact of a child’s mental illness on family members, and that they learned about effective family management strategies and access to resources. Siblings report learning coping strategies and feeling better after meeting peers with shared experiences.

## 1. Introduction

Caregivers of children with mental illness experience unique psychological challenges as well as higher rates of mental health issues compared to the general population [1,2]. Recently, a dramatic increase in mental illness has been identified in youth; currently, approximately 18–22% of youth aged 12–17 years old meet criteria for a mental disorder [2]. This produces a significant burden for caregivers as evidenced by multiple reports of heightened caregiver stress, emotional distress, and feelings of ineffectuality [2,3]. Caregivers consistently report high levels of stress and strain in caring for children with mental health needs [1]. The challenges caregivers encounter begin with a paucity of mental health resources and include barriers to accessing needed services, difficulty navigating labyrinthian care delivery systems, financial constraints, the need to advocate for their children, and experiencing stigma associated with mental illness or neurodevelopmental conditions [2,3].

In addition, siblings of children with mental health needs are significantly affected by family relational stress [4]. Studies show that in the case of children with complex developmental needs, the responsibility for caring for the child extends to next generation support providers, specifically to siblings [5]. Siblings of children with developmental disabilities, including those with behavioral challenges, often assume multiple roles, such as that of companion, protector, follower, and caregiver [6]. The literature reveals that siblings of affected children experience a mixture of stress, denial, despair, fear, guilt and impotence when confronting their sibling’s diagnosis [7] and are noted to have increased internalizing behaviors leading to greater rates of anxiety and depression. Parentification of siblings, which may have a positive effect such as enhanced compassion, may also lead to increased stress [8].

The purpose of this paper is to present an innovative, family-centered, peer-based approach to mental health care, as a novel solution to addressing caregiver and sibling stress. The Sibling Support Program: A Family-Centered Mental Health Initiative (SSP) was developed to strengthen families of youth with mental health needs by providing services through a unique combination of peer support, parent mentor involvement, and clinician-led group therapy interventions. The program offers psychoeducation on the impact of mental illness on family members, emotional support, and skill development for families across the state of Massachusetts. The SSP research questions are:

Does participation in a sibling support group increase sibling knowledge of coping skills and provide new opportunities for siblings to discuss with peers and adults their sibling’s mental health condition and its impact on their lives?

Does participation in a caregiver group increase parental knowledge and understanding, and impact their intent to employ supportive skills and resources in their families?

A description of the SSP model will be provided and preliminary results of self-report measures will be discussed with limitations and future avenues of inquiry proposed.

## 2. Literature Review: The Importance of Supporting Caregivers and Siblings

The wellbeing of caregivers and siblings often becomes overlooked in the face of more urgent needs of a child affected by mental illness. In the long term, however, caregiver well-being is critical to the endurance of the support system for a child with mental illness. Research shows that in diverse medical contexts, the impact of family-related factors on an individual’s health outcomes is comparable to or exceeds that of other risk factors [9]. A family-systems approach for treatment of children with mental illness can help to ensure that the emotional needs of caregivers, including siblings and guardians, are addressed [4]. Peer support and advocacy—hallmarks of a family-centered methodology—have become powerful therapeutic tools for families caring for children with psychiatric and neurodevelopmental disorders [2,3]. The main benefits include improved child and family management skills and function, an increased stability of living situation, improved cost-effectiveness, increased consumer and family satisfaction and improved child and family health and well-being [10].

Over the last several decades, there has been a rise in family-centered interventions that gear services toward caregivers of youth with mental illness [11]. Several broad categories of family-centered models exist, including both peer and clinician-led programs [1,12]. Peer-based programs aim to connect caregivers with similar lived experiences related to navigating healthcare delivery systems and caring for youth with mental illness [2]. Common targets of peer support interventions include psychoeducation [13,14] and skills building [15]. According to Acri et al. [3], a wide range of peer-delivered interventions for caregivers and families have been developed, but outcomes are often mixed or poorly reported. These findings are due to a variety of factors including mixed outcome measurements, diversity of intervention models, levels of training, and clinical settings. Despite these limitations, a meta-analysis of six studies showed substantial improvements in family functioning, knowledge of mental illness, and parenting skills among other reported metrics [3]. Additional studies of peer support interventions show that caregivers benefit from increased understanding of their child’s mental illness, improved management of their child’s mental health challenges, greater confidence in navigating a complicated health delivery system, and increased self-care [1,2,3,12].

In addition to caregiver education and training, there is a need for programs that focus on supporting the mental health of siblings. Programs that provide support to siblings of children with disabilities or mental illness have demonstrated positive outcomes. For example, a systematic review of interventions for siblings of children with developmental disabilities showed that the results of all the included studies demonstrated a positive impact for participant siblings [16]. Another systematic review of 14 interventions to support siblings of children with physical or mental illness concluded that such programs enhance siblings’ behavioral and emotional outcomes, leading to reduced anxiety, and improved mood and behavioral adjustment [12].

## 3. Materials and Methods

### 3.1. SSP History and Description

The SSP was developed after the Director of Sibling Support at UMass Chan Medical School (a social worker and the corresponding author in this paper) noticed three troublesome trends among families of youth admitted for psychiatric hospitalization. First, she identified that caregivers and siblings were not receiving services during and after a child’s psychiatric hospitalization, even though the events leading up to the hospitalization were often deeply traumatic (family members witnessing suicide attempts, physical assaults, police restraints, etc.). Second, she noticed that the clinicians treating the affected children were not routinely addressing the impact of the child’s illness on caregivers and typically developing siblings. Third, she observed that many caregivers of youth admitted for psychiatric treatment appeared deeply demoralized [17]. From these observations, and with determination to support families of youth in psychiatric crisis, the SSP model was born with the following objectives: provide peer support for all family members, with parents and siblings in separate groups, so they can share their stories in safe and understanding settings; incorporate parent mentors into the intervention to empower and educate parents/caregivers; and provide a training opportunity to educate mental health clinicians in family-centered care practices.

The theoretical underpinning of the SSP stems from Dr. Murray Bowen’s concept of Family Systems Theory, which posits that the family unit operates as a complex social system through which relationships, behaviors and patterns can be best understood, and that individuals are inextricably linked and interconnected within their family of origin [18,19]. Bowen believed that when one person within a family makes a change, it impacts the entire family system. Another of Bowen’s teachings that is integrated into the SSP is the technique of normalizing conflicts within a family by showing how similar conflicts exist in other families [19]. Using the Bowenian approach, the SSP views the family as a whole system, as well as identified subsystems within the family: the sibling subsystem and the parental subsystem. Focusing on family interactions within these subsystems is the foundation of the SSP.

The SSP consists of four peer support groups offered simultaneously in different rooms: a young sibling group (ages 6–11); a teen sibling group (ages 12–18); a foundational, psychoeducational group for first-time participating caregivers; and a follow-up group for returning caregivers to address ongoing stresses that impact the family unit. The sibling groups are co-facilitated by psychiatry residents and other trainees with access to clinical supervision. The caregiver groups are facilitated by trained parent mentors under the supervision of a licensed, clinical social worker. Participants are encouraged to attend groups as often as they like. The curricula for the groups are described below.

The SSP was designed with input from family members, parent mentors and clinicians, which provided, in essence, a wraparound lens through which all aspects of program delivery were examined. For example, in the planning stages, the program director assembled a SSP advisory group made up of family members to gather input on best practices. Families frequently shared that not enough clinicians understood the impact of mental illness on family members. Thus, training rotations were built into the design of the SSP, to provide hands-on opportunities for mental health trainees to work directly with impacted siblings in the group setting, and gain understanding of family-centered care strategies and skills. To date, 76 clinicians have been trained in family-centered care across SSP sites.

Other features that distinguish the SSP include involving all family members of the affected youth, as opposed to a “drop-off” sibling group model, where parents bring the sibling to a group and do not participate themselves. The SSP is also deliberate in its scheduling. The reason the SSP offers four concurrent groups simultaneously is so that all family members receive an intervention at the same time, and families are encouraged at the end of the sessions to reconnect with one another and share what they have learned. The program is intentionally offered in the early evening, a time when family members tend to be available: siblings are finished with school and extracurricular activities, and parents/caregivers are finished with the workday. Pre-COVID, we served dinner to participants, which helped to minimize stress around mealtime.

In 2011, the SSP was implemented as an IRB-approved research study on the inpatient child and adolescent psychiatry units at Cambridge Health Alliance (CHA), a public safety net hospital in Cambridge, Massachusetts. Over time, due to the success of the SSP at CHA, the program expanded to multiple sites in the community, including outpatient clinics and community centers.

The CHA site offers groups to families of youth admitted to the Child Assessment Unit (CAU) and Adolescent Assessment Unit (AAU) on a weekly basis. The community site offers groups monthly. At both sites, family members are welcome to participate in groups as often as they like. Prior to COVID-19, the peer support groups were conducted in-person. When the pandemic arrived, the SSP pivoted to online groups.

This paper reflects preliminary participant data from the IRB-approved research study at CHA, as well as participant experiences from the SSP delivered in the community. The community-based SSP is delivered through a partnership between UMass Chan Department of Psychiatry, the Massachusetts Child Psychiatry Access Program (MCPAP) and Parent/Professional Advocacy League (P/PAL), an advocacy organization for families of children and adolescents with mental health needs.

### 3.2. Recruitment and Eligibility

Participants are recruited differently at each site. At CHA, a member of the study team accesses the daily census on the child and adolescent units via EPIC and enters contact information for patient families in the SSP REDCap database [20,21], assigning unique ID codes for each participant. The SSP parent mentor calls each family to screen for eligibility and invites eligible family members to participate in the research study. Family members sign consent and assent forms when they arrive at the hospital, prior to participation.

To be eligible for the groups, siblings must be between the ages of 6–18 years and not have behavioral challenges that prevent them from engaging appropriately with peers. Parents must have more than one child, including a child with mental health needs. The sibling groups are open to all children at least 6 years of age growing up in the home alongside the affected child, including cousins, foster brothers/sisters, and stepbrothers/stepsisters. The parent groups are open to all caregivers of the affected child, such as grandparents, aunts, uncles, and other adults in caregiving roles. Parents can join the groups even if siblings do not attend, and vice versa.

At the community site, recruitment is conducted by sending flyers to community agencies, Facebook parent groups, and provider lists. Interested families call or email the SSP parent mentor to learn more about the program. The parent mentor calls each family to screen for eligibility and invites eligible family members to participate. Since the program has been delivered virtually due to the pandemic, the parent mentor emails consents and assents to participants, and they are signed electronically using a participant-specific link to REDCap [21]. Once the consent/assent has been signed, the parent mentor emails a participant-specific Zoom invitation to access the groups.

### 3.3. SSP Elective Rotations for Trainees

In addition to serving families, the SSP at both sites functions as a training opportunity for clinicians and trainees, with the goal of building capacity among providers that deliver family-centered mental health care. The sibling group at CHA is an elective rotation for psychiatry residents at Harvard Medical School. Other mental health trainees at CHA, including social work interns and psychology postdoctoral students, have joined the rotation through their own departments. Likewise, the community site serves as an elective rotation for psychiatry residents and trainees at UMass Chan Medical School. The experience of co-facilitating the sibling groups has been reported by trainees to be a highly valued learning opportunity and has provided insight into the importance of a family-centered mental health care approach. The impact of the SSP on trainees, discussed in length in a previously published paper [22], indicates that trainees who participated as sibling group facilitators were more likely to have responded that they engaged in family-centered activities during training than non-participants (*p* < 0.05), more likely to have expressed greater confidence in their family-centered care skills (*p* < 0.05), and more likely to have responded that they will practice in a family-centered way (*p* < 0.05). Lastly, trainees who served as sibling group facilitators were overwhelmingly positive about their experience with the SSP.

### 3.4. SSP Curriculum

The SSP Director designed and developed curricula for the groups. The sibling groups, co-facilitated by trainees, begin with introductions and a review of group rules, and quickly move to an activity that fosters conversation among the siblings about the experience of growing up with a brother/sister with mental health needs. Each activity includes three parts designed to help siblings process their trauma: siblings tell their stories, identify and learn coping skills, and provide support to each other. The role of the co-facilitators is also threefold: promoting a safe environment, encouraging siblings to engage with each other, and serving as knowledgeable resources. For the majority of siblings, the group setting is the first time they have met peers that share their experience. With permission from parents, siblings are given the opportunity to connect with one another outside of the group setting.

The psychoeducational group for first-time caregiver participants promotes parent-to-parent support. This group also begins with introductions and a review of group rules. The parent mentor facilitator then provides information about the sibling experience, specific parenting strategies to promote resiliency and decrease trauma, and relevant resources. Caregivers tell their stories and provide support to each other. The follow-up caregiver group, facilitated by the SSP licensed clinical social worker and parent mentor, enables returning caregivers to share ongoing struggles as part of a bonded community.

Following the groups, all the facilitators participate in a debrief session with the program director to address what went well, what could be improved, and any red flags that require follow-up with a family.

### 3.5. Data Collection

At the end of the groups, caregivers and siblings are invited to complete surveys. For in-person CHA groups, all participants are asked to complete the survey on paper prior to leaving the session. For the virtual community site, participants are emailed a unique link to complete the survey, which is then automatically associated with their record in REDCap. The survey tool asks questions about the participant’s demographics and family structure. It also asks about their perceived knowledge gain, reflection on how they felt before and after the session, any strategies they plan to utilize after the group, and their satisfaction with the group.

For survey questions with categorical responses, descriptive analyses are presented. The statistical significance of differences between in-person and virtual sessions was analyzed with chi-square analyses. Qualitative responses were coded thematically in an inductive manner, and change was assessed via a positive, neutral, or negative manner by coders for each participant. While participants were able to attend multiple sessions, only the first survey response from the first session was analyzed to assess the responses of novel participants.

Data were collected between June 2015 and March 2020 for CHA, and January 2019 and December 2021 for the community site. After March 2020, virtual surveys were used for the community site due to program delivery changes in response to COVID-19.

## 4. Results

Demographics of participating caregivers are presented in Table 1, and of participating siblings in Table 2. Caregivers participating in the program were majority female (67% CHA, 86% community). Most caregivers were aged 40–49 years, followed by 50–59 years and 30–39 years. Most participating caregivers spoke English at home (87% CHA, 99% community). White Non-Hispanic was the most common race and ethnicity indicated by participating caregivers, followed by Hispanic/Latino, then African American, then Asian, then American Indian/Alaskan Native, and then Native Hawaiian or other Pacific Islander. Caregivers were most likely to have an advanced degree or a bachelor’s degree at both CHA and in the community; however, the community caregivers were substantially more highly educated with 54% reporting an advanced degree.

Participating siblings were also more likely to be female (52% CHA, 60% community), and were most frequently aged 10–14 years (48% CHA, 58% community), followed by ages 5–9, then ages 15–18. In the virtual community groups, English was most frequently spoken in the home of participating siblings and the majority identified as White Non-Hispanic, followed by Hispanic/Latino, then African American, then Asian, then American Indian/Alaskan Native. Demographic information was largely uncollected for participating CHA siblings. One reason for the discrepancy could be that siblings at the community site were potentially receiving parental assistance to complete the virtual survey emailed to families at home, whereas CHA siblings were not given prompts for answering the demographic questions in the in-person hospital setting.

Overall, caregivers from CHA who participated in-person reported somewhat higher levels of satisfaction and were slightly more likely to report finding it helpful to meet other parents (Table 3). Across programs, 79% of caregivers reported the group very much or somewhat increased their understanding, and 59% reported it was very or somewhat helpful to meet a parent with shared experience. About 75% of caregivers participating in-person and about two-thirds of caregivers participating virtually reported learning about a new resource that they intended to pursue after the group meeting. Resources identified by caregivers largely included those related to their child’s school (e.g., IEPs and internal support resources), professional resources such as non-profits and state agencies, and parent-to-parent resources, support or resources specifically for siblings, direct programming or therapeutic support for their child with a mental health diagnosis.

As seen in Table 4, the majority of siblings (68%) indicated that it was “good to talk with people in today’s group who understand how hard it is for you to have a brother or sister with problems” and 66% would recommend the group to their peers. There was no difference in responses between siblings in the virtual or in-person programs. About half of in-person sibling group participants reported having talked to an adult about having a brother or sister with behavioral issues; a significantly greater percentage of siblings in the virtual group (84%) reported talking to an adult (X^2^ (2, *n* = 500) = 18.1, *p* < 0.05). Siblings reported learning about a range of coping skills to manage their challenging home lives, including talking to others, walking away, and using self-calming strategies such as listening to music, playing with a pet, and drawing or journaling.

There was a significant difference (X^2^ (3, *n* = 768) = 32.4, *p* < 0.05) in responses from in-person group participants and virtual group caregivers: in-person participants rated their understanding somewhat or very much increased (93%) compared with the virtual group (79%). Across in-person and virtual groups, 94% of caregivers reported they were very or somewhat satisfied with the group. In-person participants rated their satisfaction higher (78% very satisfied) compared with participants in the virtual group (61% very satisfied). The relationship between group format (in-person or virtual) and satisfaction was significant (X^2^ (3, *n* = 765) = 10.2, *p* = 0.017).

Of the participating groups, on average, about 75% more caregivers reported feeling more positive after participating in the group. About three-quarters of participating caregivers identified a new resource that they learned about from the group and intended to pursue. Caregivers reported strategies that they would try after the group such as talking more with their children that are not in mental health crisis about how they are doing, inquiring about their feelings, and spending more time and/or doing dedicated activities with these siblings. Caregivers also reported intentions to emphasize to siblings that they are not at fault and validate how having a brother/sister with a mental health condition affects them.

## 5. Discussion

There is broad consensus on the impact of mental illness on family members, as well as the impact of providing support to families of youth that struggle with mental illness, as presented in our literature review. The separate work of Tudor and Hartling, for example, shows improved outcomes for family members when the entire family receives support [12,16]. While family-centered interventions are on the rise, the SSP presents an innovative model which, to the best of our understanding, has not been developed before. What separates the SSP from other worthy family-centered interventions? As presented in the introduction, the literature about caregiver support groups suggests that most family-centered approaches involve either parent mentorship, peer support or professional support [1,3,12,16]; the SSP delivers a hybrid intervention that blends all three. The literature on the efficacy of parent mentors with lived experience suggests that parent mentors bring credibility, rapport, trust, and model advocacy skills [23]. In the SSP, the first point of contact for families is with a trained parent mentor, via a personal recruitment phone call; this establishes a relationship that is reinforced and enhanced in the parent mentor-led psychoeducation group. Additionally, the presence of clinicians in the sibling group, who bring professional expertise, ensures that siblings receive a clinical level of care. Any red flags identified by parent mentors are addressed clinically in the facilitator debrief following the sessions.

Most importantly, the SSP offers siblings and parents of children and adolescents with mental illness a place to connect with other families who are living with a similar disruption to daily life and the heart-wrenching concern about the present and future of both their child with mental illness and their whole family. In the unique setting provided by the SSP, parents and siblings participate in separate, simultaneous groups, where they are guided through discussion and activities designed to alleviate the isolation which can contribute to a sense of despair and even desperation. The majority of caregivers indicate that meeting peers who share common feelings and went through similar struggles was a positive experience. As one parent emphasized, “The biggest benefit for me is to know I’m not alone out there parenting a challenging child”.

The parent mentors facilitating the parent group share their firsthand experience raising a child with mental illness alongside the siblings. This peer-led facilitation can empower participants to reveal their own stories and take in the information gleaned by the explorations and impasses of their peers. Connecting with other parents under the guidance of the mentoring parent was described by one participant as “Survival Care—Sanity Care—those small things you can do to take care of yourself”. Another parent wrote in the survey, “I felt heard! Validated! Less alone!”.

Prior to joining the group, caregivers described feeling helpless to invent cogent and effective ways of dealing with the affected child. Once in the group, parents expressed indications of relief and identified resources they intended to use after the session. Parents routinely expressed feeling hopeful despite the extreme duress they experienced in their home lives. One parent was “comforted by a few more tools under my belt for helping my daughter process her feelings”. Another wrote, “I felt more confident that we have the tools to support them (my children) and each other”.

Both the parents and the siblings indicated feelings of emotional relief, as seen in the positive “before and after” trends in the data. In the parent group, the awareness of shared experience, absence of blame and the sharing and discussion of actual concrete, practical directions were identified in these preliminary data as valuable components of the program. The majority of parents were able to identify resources they intended to pursue to help themselves and their families. The majority of caregivers reported an increased understanding of the experience of siblings. The majority also reported intention to make changes in their parenting strategies that included paying more attention to siblings, communicating more effectively and validating the sibling’s experiences.

Of interest is that these outcomes, while positive, appeared to mildly diminish when in-person groups were switched to a virtual platform in response to the emergence of the COVID-19 global pandemic. This was possibly a reflection of additional family stress brought by the pandemic, as well as logistical stress that can accompany remote participation.

Preliminary data from the sibling group indicate a similar level of relief among children and teens. Almost half of the children in the sibling group reported they had not spoken to an adult about their experience with a mentally ill sibling. This suggests that nearly half of the siblings perceived the subject was not to be raised, and—in keeping with the literature on sibling experiences of stress, denial, despair, fear, guilt and impotence [7]—one might assume this may be associated with shame about their family should others know what was harbored there. Siblings who had been keeping secrets about scary things happening at home found they were not alone. One sibling reported, “I like this group because you meet other people who have the same issues and sometimes you feel alone dealing with this stuff”. The group setting can reveal that other children, younger and older as well as their own age, live with the same set of challenges. For example, they indicated the challenges of navigating in a home where the fragility or volatility of a brother or sister with mental illness dictates all of the activity and reactivity of a household.

The sibling group activities, designed to help siblings first recognize the elements of their shared experience and then to articulate those experiences and the attendant feelings, appeared to meet those goals in these preliminary data. The majority of siblings reported it was “good” to have met a peer who understood what they had endured. “I liked sharing my fidgets with everyone. I was happy to meet new kids”, one young sibling said. The majority of siblings reported they would recommend the group to others in their shoes. Siblings also responded that they left the group with strategies that they could use to try to help themselves when upset with their sibling, including communicating more about their feelings, physically distancing themselves from their sibling when needed, and using calming techniques.

The preliminary data suggest that the positive immediate responses to the SSP among participants are attributable to the benefits of meeting peers with lived experience and being introduced to accessible coping skills, parenting strategies and resources, bolstered by the expertise of the professionals operating as teams. Including peers in team work to foster change and to make the unbearable tolerable shows promise as an important ingredient in the recipe for moving forward.

## 6. Limitations

The preliminary data presented were collected from voluntary surveys completed at the end of the respective group sessions. Because pre–post intervention data were not collected, a few questions ask participants to reflect on the period before participation which may be subject to some bias. Therefore, the program and the preliminary data collected may both be subject to several biases including selection and assessment bias. Additionally, in response to the COVID-19 pandemic, the previously in-person program pivoted to a virtual platform. As a result, response rates to emailed surveys dropped significantly. This limited the amount of data collected and possibly impacted the conclusions that could be drawn from the preliminary data obtained.

Questions in the survey asked about what caregivers and siblings intend to do but were not able to assess which strategies were actually used after participation. The survey data in this paper were collected after participation in one session, though some caregivers and siblings participated in multiple sessions and that may have had a more meaningful impact on their families. Control caregivers or siblings were not included in this analysis.

The demographic data of participants across the various groups reveal that participants were from largely homogenous racial groups with the majority identifying as white with the next largest minority groups being Hispanic, Black, and Asian. While efforts were made to invite and retain members of diverse backgrounds, the program materials and groups were provided in English only. It is also worth noting that most participants represented higher socioeconomic backgrounds as suggested by the high percentage of parents having earned degrees in higher education. Further efforts could be made to find, invite, and include participants from a wider range of socioeconomic, ethnic, and racial backgrounds.

Finally, some participants faced barriers to participation. Pre-COVID, although attendance was incentivized by offering a pizza dinner, childcare and transportation created barriers for some participants, especially among lower income families. Further efforts could be made to remove these barriers including fundraising to pay for transportation vouchers and childcare for in-person groups. When the program switched to virtual telehealth platforms during COVID, several modifying factors might have inhibited participation, including the distractions of logging in from home, a lack of privacy at home, and a lack of access or poor connection to the internet.

## 7. Conclusions

The SSP is a novel, peer-based, family-centered intervention that provides support to parents, caregivers, and siblings of children with mental illness. The intervention combines psychoeducation, a family-centered forum to process experiences in parallel with other family members, and information about community and professional resources. The program’s uniqueness stems from a blend of peer support, psychoeducation, behavioral interventions, and parenting modeling using parent mentors and clinician trainees, including psychiatry residents, social work interns and psychology interns. Preliminary data gathered from participants suggest caregivers and youth found the program’s resources to be helpful, and participant surveys reflect increases in knowledge, access to resources, and ideas for coping with challenging family dynamics. These findings are in keeping with the positive yet mixed outcomes that have been reported in the literature from other interventions and provide another model through which to support family members of youth experiencing mental illness. Preliminary findings suggest initial perceived benefit among participants from the combined components of this programmatic model. Further investigation is needed to establish and further characterize the clinical impacts of this family-centered model.

## Figures and Tables

**Table 1:** Caregiver participant demographics.

Demographic Factor		In-Person (*n* = 698)		Virtual (*n* = 70)		Total (*n* = 768)	
		*n*	%	*n*	%	*n*	%
Gender	Female	466	67%	60	86%	526	68%
	Male	226	32%	10	14%	236	31%
	Other	6	1%	0	0%	6	1%
Age	Under 20	2	0.3%	0	0%	2	0.3%
	20–29 years	27	4%	0	0%	27	4%
	30–39 years	157	22%	9	13%	166	22%
	40–49 years	309	44%	46	66%	355	46%
	50–59 years	160	23%	14	20%	174	23%
	Over 60 years	39	6%	1	1%	40	5%
	Not reported	4	1%	0	0%	4	1%
Language Spoken at Home	English	606	87%	69	99%	675	88%
	Haitian Creole	8	1%	0	0%	8	1%
	Other	37	5%	1	1%	38	5%
	Portuguese	9	1%	0	0%	9	1%
	Spanish	35	5%	0	0%	35	5%
	Not reported	3	0.4%	0	0%	3	0.4%
Race/Ethnicity	African American/Black	66	9%	3	4%	69	9%
	American Indian/Alaskan Native	9	1%	0	0%	9	1%
	Asian	35	5%	3	4%	38	5%
	Hispanic/Latino Spanish	88	13%	4	6%	92	12%
	I don’t know	2	0.3%	0	0%	2	0%
	Native Hawaiian or other Pacific Islander	2	0.3%	1	1%	3	0%
	Other	20	3%	1	1%	21	3%
	White	493	71%	59	84%	552	72%
Highest Level of Education	Advanced degree	170	24%	38	54%	208	27%
	Bachelor’s Degree (4 year)	162	23%	22	31%	184	24%
	Associate Degree (2 year)	61	9%	5	7%	66	9%
	Some College or Training Program	144	21%	2	3%	146	19%
	High School/GED	90	13%	2	3%	92	12%
	Some High School	38	5%	0	0%	38	5%
	Other	27	4%	0	0%	27	4%
	Not reported	6	1%	1	1%	7	1%

**Table 2:** Sibling participant demographics *.

Demographic Factor		In-Person (*n* = 455)		Virtual (*n* = 50)		Total (*n* = 505)	
		*n*	%	*n*	%	*n*	%
Gender	Female	236	52%	30	60%	266	53%
	Male	216	47%	17	34%	233	46%
	Other	1	0.2%	2	4%	3	1%
	Not reported	2	0.4%	1	2%	3	1%
Age (years)	5–9	156	34%	12	24%	168	33%
	10–14	217	48%	29	58%	246	49%
	15–19	81	18%	6	12%	87	17%
Language Spoken at Home	English	46	10%	49	98%	95	19%
	Haitian Creole	0	0%	0	0%	0	0%
	Other	1	0%	0	0%	1	0%
	Portuguese	1	0%	0	0%	1	0%
	Spanish	4	1%	0	0%	4	1%
	Not reported	403	89%	1	2%	404	80%
Race/Ethnicity	Hispanic/Latino Spanish	15	3%	4	8%	19	4%
	American Indian/Alaskan Native	1	0%	50	100%	51	10%
	African American/Black	6	1%	10	20%	16	3%
	White	28	6%	36	72%	64	13%
	Asian	3	1%	7	14%	10	2%
	Native Hawaiian or other Pacific Islander	0	0%	0	0%	0	0%
	Other Race/ethnicity	2	0%	0	0%	2	0%
	I don’t know race/ethnicity	6	1%	0	0%	6	1%

* As mentioned in the Results section, please note that demographic information was largely uncollected for participating CHA siblings. One reason for the discrepancy could be that siblings at the community site were potentially receiving parental assistance to complete the virtual survey emailed to families at home, whereas CHA siblings were not given prompts for answering the demographic questions in the in-person hospital setting.

**Table 3:** Caregiver knowledge gain, satisfaction and perceived change.

Response	In-Person (*n* = 698)		Virtual (*n* = 70)		Total (*n* = 768)	
	*n*	%	*n*	%	*n*	%
How much has participating in today’s group increased your understanding of the sibling experience? *						
Very Much	445	63.8%	24	34.3%	469	61%
Somewhat	204	29.2%	31	44.3%	235	31%
A little	45	6.4%	15	21.4%	60	8%
Not at all	4	0.6%	0	0.0%	4	1%
Was it helpful to meet another parent who shares your experience?						
Very Helpful	466	66.8%	31	44.3%	497	65%
Somewhat helpful	155	22.2%	10	14.3%	165	21%
A little helpful	53	7.6%	5	7.1%	58	8%
Not at all helpful	5	0.7%	0	0.0%	5	1%
How satisfied were you with today’s group? *						
Very Satisfied	541	77.5%	43	61.4%	584	76%
Somewhat satisfied	136	19.5%	23	32.9%	159	21%
A little satisfied	17	2.4%	4	5.7%	21	3%
Not at all satisfied	1	0.1%	0	0.0%	1	0%
Categories of things caregivers (CGs) indicate they will do differently after the group to support siblings:						
Communicate	255	37%	21	30%	276	36%
Attend to/check in with sibling	122	17%	10	14%	132	17%
Assure/validate sibling	123	18%	18	26%	141	18%
Instruct or get support for sibling	147	21%	22	31%	169	22%
Self-care	0	0%	0	0%	0	0%
Other	74	11%	14	20%	88	11%
No Answer	117	17%	7	10%	124	16%
Resources learned about in the group that caregivers intend to pursue:						
School/IEP	38	5%	10	14%	48	6%
Non-profit/family support	250	36%	20	29%	270	35%
Sibling support	92	13%	7	10%	99	13%
Direct programming	28	4%	4	6%	32	4%
Other	187	27%	12	17%	199	26%
Nothing identified	178	26%	23	33%	201	26%
* = significant difference between virtual and in-person groups with *p* < 0.05						
Before coming to today’s group, I felt:			Now that the group is over, I feel:			
CG in-person before			CG in-person after			
Positive	50	7%	Positive	586	84%	
Neutral	170	24%	Neutral	45	6%	
Negative	412	59%	Negative	13	2%	
No answer	66	9%	No answer	54	8%	
CG virtual before			CG virtual after			
Positive	3	4%	Positive	52	74%	
Neutral	33	47%	Neutral	4	6%	
Negative	23	33%	Negative	3	4%	
No answer	11	16%	No answer	11	16%	

**Table 4:** Sibling knowledge gain, satisfaction and perceived change.

Response	In-Person (*n* = 455)		Virtual (*n* = 50)		Total (*n* = 505)	
	*n*	%	*n*	%	*n*	%
Have you ever talked to an adult about having a brother or sister with problems? *						
Yes	234	51%	41	82%	275	54%
No	148	33%	6	12%	154	30%
Not sure	69	15%	2	4%	71	14%
Was it good to talk with people in today’s group who understand how hard it is for you to have a brother or sister with problems?						
Yes	305	67%	36	72%	341	68%
Maybe	128	28%	11	22%	139	28%
No	20	4%	2	4%	22	4%
Would you recommend this group to other kids who have a brother or sister like yours?						
Yes	301	66%	31	62%	332	66%
Maybe	126	28%	14	28%	140	28%
No	26	6%	4	8%	30	6%
What did you learn today about what to do when you are upset with your brother or sister?						
Talk to someone	46	10%	4	8%	50	10%
Walk away	106	23%	18	36%	124	25%
Calming coping strategies	132	29%	14	28%	146	29%
Try to understand	57	13%	1	2%	58	11%
Other	40	9%	4	8%	44	9%
Nothing identified	91	20%	12	24%	103	20%
The most helpful part of the group for siblings						
Talking	181	40%	12	24%	193	38%
Making friends	71	16%	27	54%	98	19%
Learning what to do	58	13%	5	10%	63	12%
Food	50	11%	0	0%	50	10%
Other	47	10%	2	4%	49	10%
Nothing identified	51	11%	6	12%	57	11%
* = significant difference between virtual and in-person groups with *p* < 0.05						
Sibling group in-person before			Sibling group in-person after			
Positive	120	26%	Positive	375	82%	
Neutral	89	20%	Neutral	51	11%	
Negative	234	51%	Negative	19	4%	
No answer	12	3%	No answer	10	2%	
Sibling group virtual before			Sibling group virtual after			
Positive	6	12%	Positive	38	76%	
Neutral	12	24%	Neutral	2	4%	
Negative	27	54%	Negative	4	8%	
No answer	5	10%	No answer	6	12%	

## Data Availability

Study data were collected and managed using REDCap electronic data capture tools hosted at UMass Chan Medical School. REDCap (Research Electronic Data Capture) is a secure, web-based software platform designed to support data capture for research studies, providing (1) an intuitive interface for validated data capture; (2) audit trails for tracking data manipulation and export procedures; (3) automated export procedures for seamless data downloads to common statistical packages; and (4) procedures for data integration and interoperability with external sources. Data can be made available upon request. Please direct communication to the corresponding author.
